# 

**Published:** 1818-03

**Authors:** 


### CATALOGUE OF BOOKS.

Aphorisms, illustrating Natural and Difficult Cases of Accouchement, Uterine Hemorrhage, and Puerperal Peritonitis. By Andrew Blake, M.D. Small 8vo.

Memoirs and Reports, on the Efficacy of Sulphurous Fumigation in the Treatment of Diseases of the Skin, Joints, and Glandular System, Chronic Rheumatism, Gout, Paralytic Affections, &c. &c. From the French, (published by order of that government,) of J. C. Gales, M.D. of the Faculty of Medicine of Paris, Corresponding Member of the Medical Society of Toulouse, &c. By Rees Price, Member of the Royal College of Surgeons, London.

Observations on some Important Points in the Practice of Military Surgery, and in the Arrangement and Police of Hospitals; illustrated by Cases and Dissections. By John Hennen, Deputy Inspector of Military Hospitals. 8vo. 4s.

A Letter to the Commissioners for Transports and Sick and Wounded Seamen, on the Non-contagious Nature of the Yellow-Fever; and containing Hints to Officers for the Prevention of this Disease among Seamen. By James Veitch, M D. 8vo. 7s.

A Treatise on the Principal Diseases of the Eyes. By Antonio Scarpa. Illustrated by Engravings. Translated from the Italian, with Notes, by James Briggs. Second edition, considerably enlarged. 8vo. 14s.

---

### *NOTICES TO CORRESPONDENTS.*

### *Letter from Dr. Renwick, of Liverpool, to the Editors.*

GENTLEMEN.—I will be obliged to you to correct, in the next Number of your Journal, the following errors of the press:—Page 164, line 20, for *Mr.* read *Dr.* Brandreth; same page, line 21, for *Bickerstaffe* read *Bickersteth.* Page 165, line 21, read *she appears to waste in the right side and in the right breast;* same page, line 22, for *enormous* read *anasarcous;* also, the same page, line 33, for *Allison* read *Alanson;* at least, I think you must mean my friend, Mr. Edward Alanson, formerly a leading surgeon in Liverpool, and the author of a Treatise upon Amputation. Mr. A. resided at Wavertree, near Liverpool, where he has retired to enjoy the result of an active and well-spent life, the *otium cum dignitate*, to which he is so well entitled. You have quoted a note from my narrative of Miss M'Avoy's case, in page 116 of your Journal of this month, respecting the person to whom the Blind Asylum of Liverpool owes its origin.—I have named the Rev. Henry Dunnett, as the person generally supposed to have been the first promoter of this institution. Mr. Edward Rushton reports his father to have been the first who gave the idea of this establishment. Mr. G. Rushton's account of this circumstance will be found in the Liverpool Mercury of the 12th of December last, and one of the following numbers. I am, gentlemen, your's, &c.

*Liverpool; Feb.* 4, 1818. THOMAS RENWICK.

ALPHABETICUS *will find we have already noticed* Dr. MARTIN's *book. If the writer will lend us a copy, we will examine what further use can be made of it.* Mr. HALL's *work on Semaiology will be noticed, when we have less temporary matter on hand. The favours from* Deputy-Inspector HENNEN *shall be duly attended to.* PROPTER QUOD *ought to know, that we never admit anonymous remarks on the favours of gentlemen who honour us with their proper names; he must, therefore, permit us to shorten his paper, or it must be signed by his name.*

